# Human Prion Diseases in the United States

**DOI:** 10.1371/journal.pone.0008521

**Published:** 2010-01-01

**Authors:** Robert C. Holman, Ermias D. Belay, Krista Y. Christensen, Ryan A. Maddox, Arialdi M. Minino, Arianne M. Folkema, Dana L. Haberling, Teresa A. Hammett, Kenneth D. Kochanek, James J. Sejvar, Lawrence B. Schonberger

**Affiliations:** 1 Division of Viral and Rickettsial Diseases, National Center for Zoonotic, Vector-borne and Enteric Diseases, Centers for Disease Control and Prevention (CDC), U.S. Department of Health and Human Services (USDHHS), Atlanta, Georgia, United States of America; 2 Division of Vital Statistics, National Center for Health Statistics, Centers for Disease Control and Prevention (CDC), U.S. Department of Health and Human Services (USDHHS), Hyattsville, Maryland, United States of America; University of Kent, United Kingdom

## Abstract

**Background:**

Prion diseases are a family of rare, progressive, neurodegenerative disorders that affect humans and animals. The most common form of human prion disease, Creutzfeldt-Jakob disease (CJD), occurs worldwide. Variant CJD (vCJD), a recently emerged human prion disease, is a zoonotic foodborne disorder that occurs almost exclusively in countries with outbreaks of bovine spongiform encephalopathy.

This study describes the occurrence and epidemiology of CJD and vCJD in the United States.

**Methodology/Principal Findings:**

Analysis of CJD and vCJD deaths using death certificates of US residents for 1979–2006, and those identified through other surveillance mechanisms during 1996–2008. Since CJD is invariably fatal and illness duration is usually less than one year, the CJD incidence is estimated as the death rate. During 1979 through 2006, an estimated 6,917 deaths with CJD as a cause of death were reported in the United States, an annual average of approximately 247 deaths (range 172–304 deaths). The average annual age-adjusted incidence for CJD was 0.97 per 1,000,000 persons. Most (61.8%) of the CJD deaths occurred among persons ≥65 years of age for an average annual incidence of 4.8 per 1,000,000 persons in this population. Most deaths were among whites (94.6%); the age-adjusted incidence for whites was 2.7 times higher than that for blacks (1.04 and 0.40, respectively). Three patients who died since 2004 were reported with vCJD; epidemiologic evidence indicated that their infection was acquired outside of the United States.

**Conclusion/Significance:**

Surveillance continues to show an annual CJD incidence rate of about 1 case per 1,000,000 persons and marked differences in CJD rates by age and race in the United States. Ongoing surveillance remains important for monitoring the stability of the CJD incidence rates, and detecting occurrences of vCJD and possibly other novel prion diseases in the United States.

## Introduction

Prion diseases, or transmissible spongiform encephalopathies (TSEs), are a family of rare, progressive, invariably fatal, neurodegenerative disorders that affect both humans and animals [1], [2]. Prions are agents that are capable of inducing abnormal folding of the cellular prion protein through a mechanism that remains a matter of debate.^3^ They are the purported unconventional causative agents of this family of diseases that includes classic Creutzfeldt-Jakob disease (CJD) and variant CJD (vCJD) in humans [1], [2], [4].

The cause of the most common form of CJD, sporadic CJD, is unknown, but a widely held hypothesis is that it may result from spontaneous conversions of the normal prion protein to its pathogenic form without the necessity of an environmental source of infection [5]–[8]. CJD is generally characterized by a rapidly progressive dementia in older adults and usually death occurs within 1 year of illness onset [9], [10]. In contrast, vCJD occurs almost exclusively in persons less than 55 years of age. A median illness duration of 13–14 months from symptom onset until death follows an incubation period that has been estimated to be about 17 years [1], [2], [11]. Clinical and epidemiological differences are important in distinguishing cases of CJD from vCJD, but neuropathologic study of brain tissue is required for confirmation [1], [2], [12]. The distinction between CJD and vCJD is important, particularly for public health practitioners, because strong evidence suggests that vCJD, unlike classic CJD, can be transmitted person-to-person by blood transfusion and acquired by ingestion of food contaminated with the agent of bovine spongiform encephalopathy (BSE, commonly known as “mad cow” disease) [4], [13]–[16].

In the United States, monitoring the occurrence of prion diseases relies on different surveillance mechanisms, the most systematic and cost effective of which consists of periodic analyses of the national multiple cause-of-death data. The present study describes the epidemiology of CJD and the occurrence of vCJD in the United States.

## Methods

Death records with CJD listed as a cause of death were obtained from the US national multiple cause-of-death data maintained by the National Center for Health Statistics (NCHS), Centers for Disease Control and Prevention (CDC) [17]. Records with the CJD International Classification of Disease, Ninth Revision (ICD-9) code of 046.1 for 1979–1998 and the CJD ICD, Tenth Revision (ICD-10) code of A81.0 for 1999–2006 were selected annually for analysis [18], [19]. The multiple cause-of-death data consist of all death certificate data, except for 1981 and 1982 data which are nationally representative weighted samples [20], [21].

In addition to using the multiple cause-of-death data to identify CJD deaths, some CJD records were added and CJD-coded records removed from the CJD deaths dataset as a result of information obtained from other surveillance efforts as of June 1, 2009. These efforts, included investigations, in collaboration with state health authorities, of cases of possible vCJD reported by clinicians to CDC directly or indirectly through local or state public health officials or possibly through prion disease surveillance personnel from outside the United States. These efforts also included routinely collecting and reviewing medical records of reported cases of suspected or diagnosed cases of CJD in patients under 55 years of age identified through the multiple cause-of-death data, or reported to CDC by health department personnel, members of the CJD Foundation, or staff at the National Prion Disease Pathology Surveillance Center (NPDPSC). In the present study, the term CJD or classic CJD refers to the forms of human prion disease that are not variant CJD unless otherwise specified.

Beginning with the 1999 surveillance year, the CJD death dataset includes the review of information from computerized literal text cause-of-death data, the first national application of such data. These literal text data help to overcome a problem introduced in 1999 by the new CJD-related ICD-10 coding rule that would otherwise lead to undercounts of reported CJD deaths. Creutzfeldt-Jakob disease was considered among a group of illnesses classified as a “rare event” that required additional checking for possible errors. However, under the new coding scheme, coders were instructed to code CJD with a duration of ≥1 year as “sequelae of other specified infectious diseases” (ICD-10 code B94.8) and not as CJD (A81.0) which prevented identification of CJD as a reported cause of death [19] (this coding rule has subsequently been changed beginning in 2007 such that these deaths will be coded to A81.0). To compensate for this latter limitation in the surveillance of CJD, deaths beginning in 1999 reported with CJD were identified utilizing a search of the literal text for CJD and CJD-related terms on death certificate records. This search used SuperMICAR (Mortality Medical Indexing, Classification, and Retrieval system), a component of the Mortality Medical Data System, an automated data system for coding cause of death [22]. All the information found on the death certificate, including literal text, is entered into this system. The data are then processed (e.g., dividing terms, replacing words with synonyms, dropping unnecessary words, and rearranging words) and matched to a MICAR dictionary. An input file is generated which assigns ICD-10 codes to the cause-of-death data. The SuperMICAR allows for querying and sub-setting of records based on literal text. It is this functionality that was used to search for CJD-related terms. In 1999, 18 states had SuperMICAR data available to NCHS; this number increased to 29 states in 2000, 39 states in 2001, 49 states in 2002, and all states beginning in 2003.

Records identified from the SuperMICAR literal search of CJD-related terms that did not have an ICD-10 code of A81.0 were manually reviewed to verify CJD as the probable listed cause of death. Non-CJD coded death records found to have a CJD-related literal text through the SuperMICAR process were added to the CJD surveillance database. In addition, records with an ICD-10 code of A81.0 were matched with the SuperMICAR data and manually reviewed for possible miscoding as CJD; records were then excluded if information was found that contradicted a prion disease diagnosis.

The incidence of CJD was estimated by using death rates because CJD is invariably fatal with over 85% of cases dying within one year of onset [23], [24]. The number of deaths with CJD and the corresponding US census population estimates for each year were used to calculate annual and average annual CJD age-specific and age-adjusted death rates per 1,000,000 persons [25]. Average annual age-adjusted incidence rates for the overall population and by sex, race, and geographic region (standard census regions Northeast, Midwest, South, and West) [26] were calculated by the direct method using the projected 2000 population for the United States [27], [28]. Risk ratios (RRs) and age-adjusted RRs were calculated by using Poisson regression analysis with approximate large-sample 95% confidence intervals (CIs).

Deaths with CJD were also examined for records with diseases associated with increased exposure to blood or blood products, specifically hereditary factor VIII (hemophilia A; ICD-9 code 286.0 and ICD-10 code D66), hereditary factor IX (hemophilia B; codes 286.1 and D67), thalassemia (codes 282.4 and D56), and sickle cell disease (codes 282.6 and D57) [18], [19].

## Results

### Creutzfeldt-Jakob Disease

**Figure 1:**
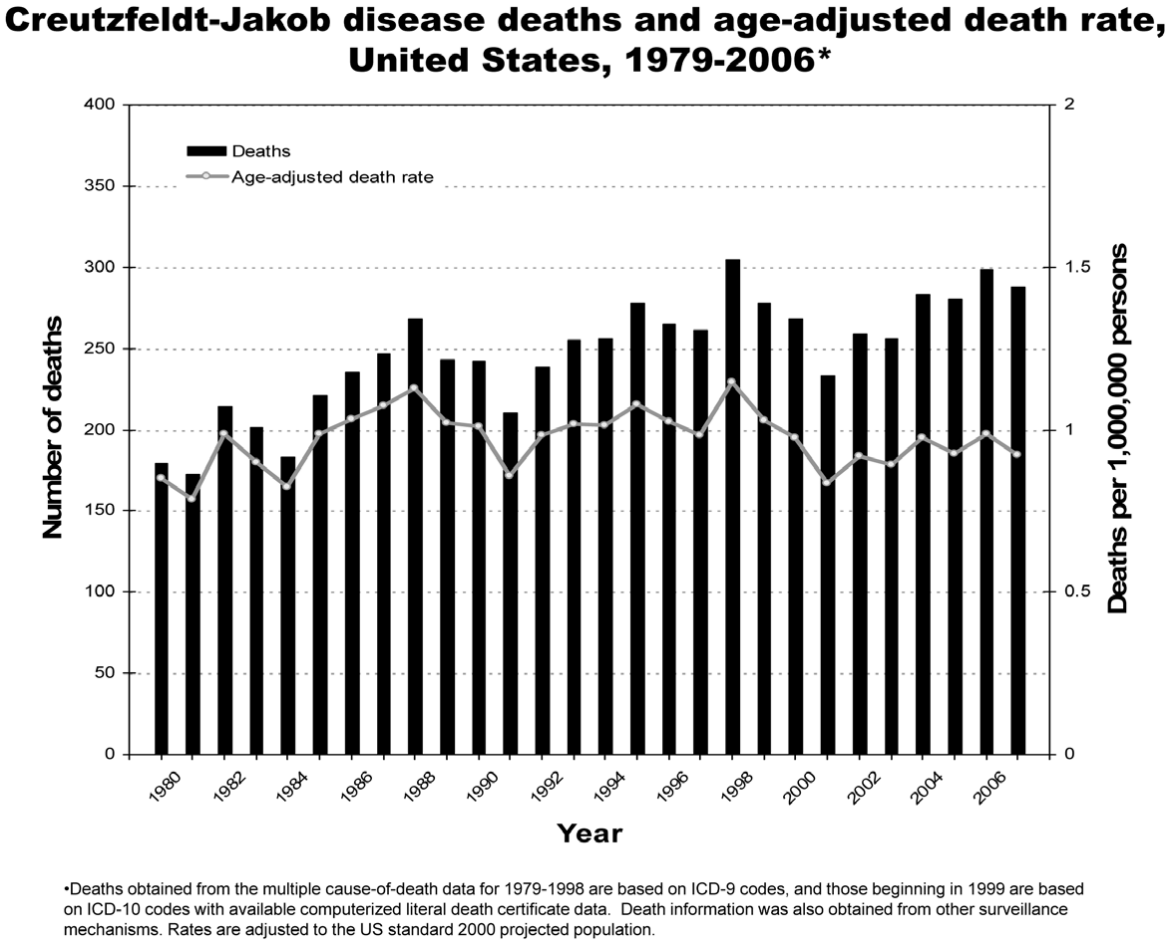
Creutzfeldt-Jakob disease deaths and age-adjusted death rates, United States, 1979–2006.

**Figure 2:**
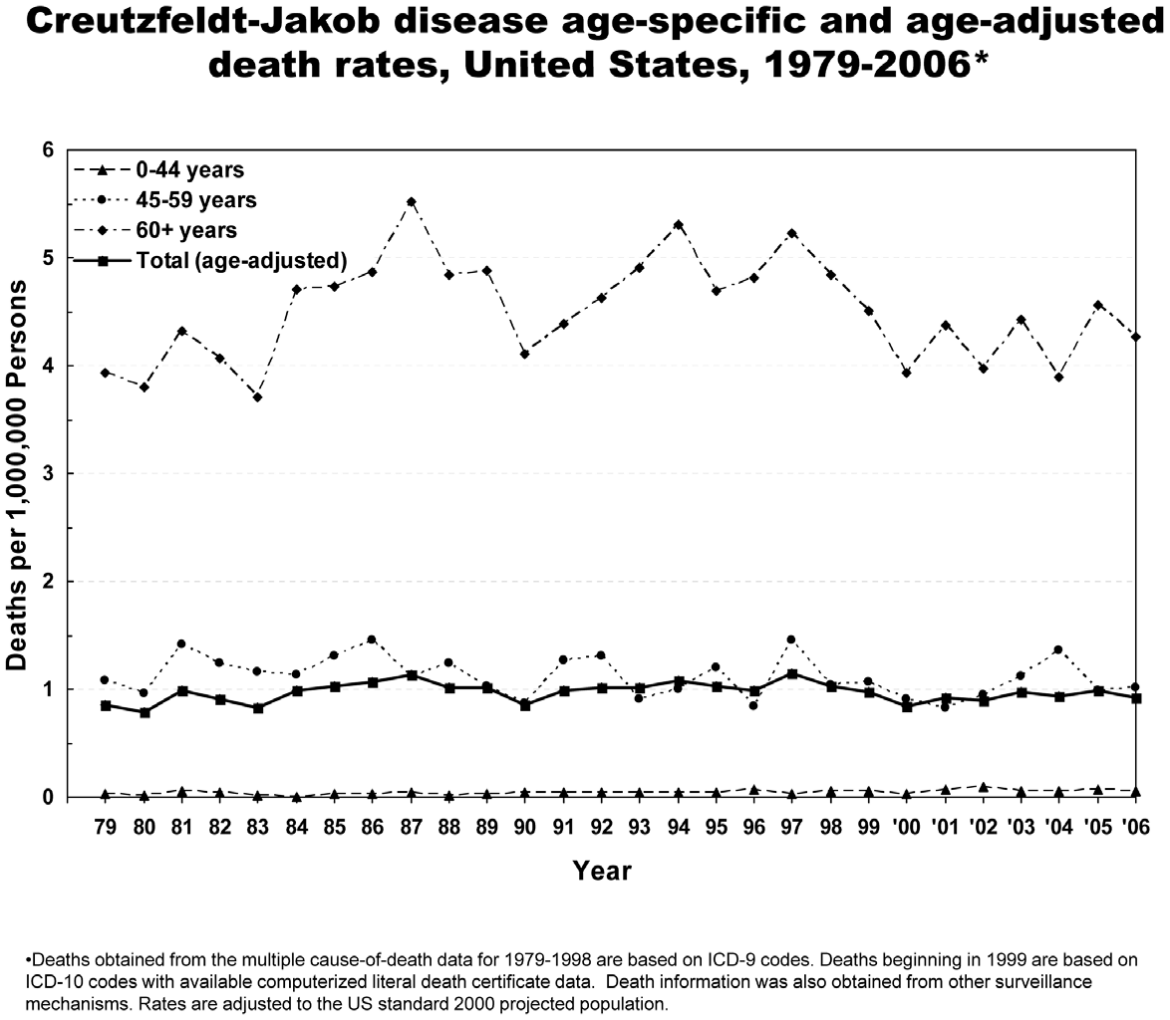
Creutzfeldt-Jakob disease age-specific and age-adjusted death rates, United States, 1979–2006.

From 1979 through 2006, 6,917 deaths due to CJD were reported in the United States (Table 1, Figure 1). CJD was recorded as the underlying cause for 83% of the deaths. An average of approximately 247 deaths occurred annually, ranging from 172 in 1980 to 304 in 1997. During 2003–2006, the years for which all states submitted literal text, an average of 288 prion disease deaths were reported annually. The annual incidence of CJD has remained relatively stable from 1979–2006 in the United States at approximately 1 case per 1,000,000 persons (Figure 1). The incidence was higher among the older population (Figure 2). The average annual incidence for persons ≥65 years of age was 4.8 per 1,000,000 persons.

**Table 1:** Creutzfeldt-Jakob disease death rates and deaths, United States, 1979–2006.*

Characteristics	Death Rate (95% CI) †	Risk Ratio (95% CI) †	Number of Deaths (%)
Age group			
<55 years	0.14 (0.13–0.15)	reference	781 (11.3)
≥55 years ‡	3.97 (3.87–4.07)	29.1 (27.0–31.4)	6136 (88.7)
Sex			
Female	0.92 (0.89–0.95)	reference	3635 (52.6)
Male ‡	1.05 (1.01–1.09)	1.2 (1.2–1.3)	3282 (47.4)
Race			
Black	0.40 (0.35–0.45)	reference	254 (3.7)
White ‡	1.04 (1.01–1.07)	2.7 (2.4–3.0)	6541 (94.6)
Other ‡	0.61 (0.50–0.73)	1.5 (1.2–1.9)	121 (1.7)
Region			
South	0.84 (0.81–0.88)	reference	2103 (30.4)
Northeast ‡	1.08 (1.03–1.13)	1.2 (1.2–1.3)	1656 (24.0)
Midwest ‡	1.04 (0.99–1.09)	1.2 (1.1–1.2)	1793 (25.9)
West ‡	0.98 (0.93–1.04)	1.2 (1.1–1.2)	1363 (19.7)
Total	0.97 (0.95–0.99)		6917

* Deaths obtained from the multiple cause-of-death data for 1979–1998 are based on ICD-9 codes and those beginning in 1999 are based on ICD-10 codes with available computerized literal death certificate data. Death information was also obtained from other surveillance mechanisms. One death is missing both race and region; and one death is missing region.

† Death rates expressed per 1,000,000 persons in corresponding group. Death rates and risk ratios are age-adjusted for total, sex, race, and region. The 95% confidence intervals (CIs) are given.

‡ p<0.001 for comparison of death rate to that of the referent group.

**Figure 3:**
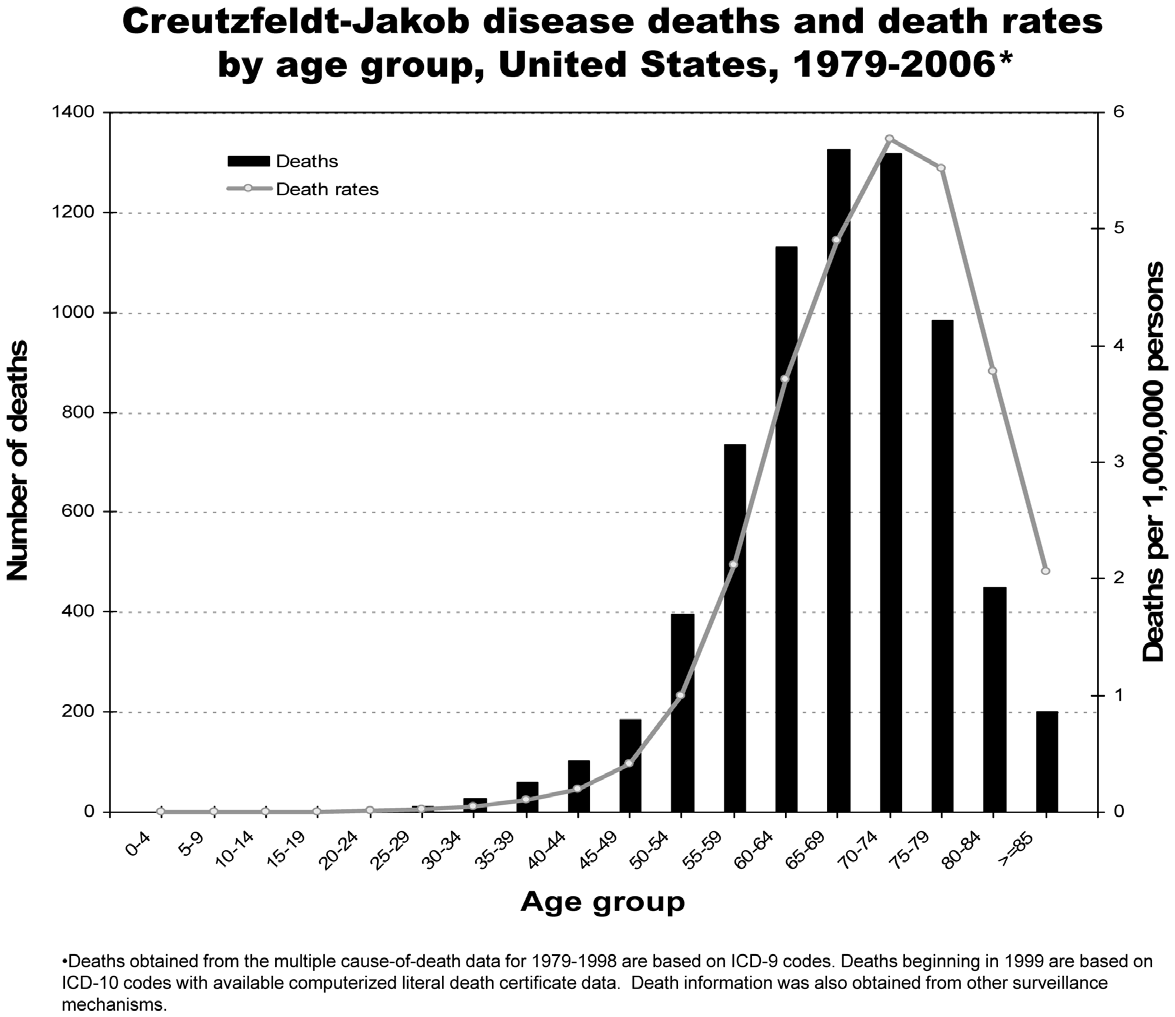
Creutzfeldt-Jakob disease deaths and death rates by age group, United States, 1979–2006.

The median age at death for the study period was 68 years (range 21 to 97; Figure 3). The annual median age ranged from 64 to 70 years of age. Most deaths occurred among persons 60–79 years of age, and the highest death rates were among those 65–79 years of age (Figure 2, Figure 3). Fifteen decedents were <30 years of age. Of these, five were sporadic cases, four were human growth hormone-associated cases, three were dura mater-associated cases, and two were familial cases due to genetic mutation on the prion protein gene; the CJD classification of one young case from 1981 was unknown. Therefore, at least 60% of the classified cases <30 years of age are attributable to iatrogenic exposure or a genetic mutation. No CJD-related deaths were reported with hereditary factor VIII (hemophilia A), hereditary factor IX (hemophilia B), thalassemia, or sickle cell disease.

A majority (52.6%) of the CJD cases occurred in females, but the average annual age-adjusted incidence for males was higher than that for females (RR = 1.2, 95% CI = 1.2–1.3; Table 1). However, this higher rate for males was only seen among those ≥60 years of age and it increased relative to females as age increased (RR = 1.2 for 60–69 year age group and RR = 1.4 for ≥80 years group; Table 2). The median age at death for males and females was 67 and 68 years, respectively.

**Table 2:** Creutzfeldt-Jakob disease deaths and death rates by age group, sex, race, and region, United States, 1979–2006.*

Age group (years)	Sex				Race†						Region†							
	Male		Female		White		Black		Other		Northeast		Midwest		South		West	
	Deaths	Rate	Deaths	Rate	Deaths	Rate	Deaths	Rate	Deaths	Rate	Deaths	Rate	Deaths	Rate	Deaths	Rate	Deaths	Rate
0–49	190	0.1	196	0.1	347	0.1	26	0.04	12	0.05	87	0.1	96	0.1	125	0.1	77	0.1
50–59	530	1.5	600	1.6	1049	1.6	56	0.7	25	0.9	259	1.7	261	1.5	361	1.4	248	1.6
60–69	1224	4.6	1229	4.0	2329	4.6	78	1.5	46	2.7	573	4.7	653	4.8	753	3.7	474	4.3
70–79	1072	6.3	1227	5.2	2200	6.0	75	2.3	24	2.5	574	6.5	609	6.2	672	4.7	444	5.8
≥80	266	3.7	383	2.6	616	3.1	19	1.2	14	3.6	163	3.4	174	3.1	192	2.6	120	3.0

* Deaths obtained from the multiple cause-of-death data for 1979–1998 are based on ICD-9 codes, and deaths beginning in 1999 are based on ICD-10 codes with available computerized literal death certificate data. Death information was also obtained from other surveillance mechanisms. Death rates expressed per 1,000,000.

† One death is missing both race and region; and one death is missing region.

The vast majority (94.6%) of deaths were among whites (Table 1). The average annual age-adjusted incidence rate was more than two and a half times higher for whites than that for blacks (1.04 and 0.40, respectively; RR = 2.7, 95% CI = 2.4–3.0). The average annual age-adjusted incidence rates were highest in the Northeast region and lowest in the South region. The higher incidence rate for whites compared to that for blacks were seen in each of the regions (Table 3).

**Table 3:** Creutzfeldt-Jakob disease deaths and death rates by age group, region and race, United States, 1979–2006.*

Age group (years)	Northeast				Midwest				South				West			
	White		Black		White		Black		White		Black		White		Black	
	Deaths	Rate	Deaths	Rate	Deaths	Rate	Deaths	Rate	Deaths	Rate	Deaths	Rate	Deaths	Rate	Deaths	Rate
0–49	77	0.1	9	0.1	91	0.1	2	<0.1	113	0.1	12	0.03	66	0.1	3	0.04
50–59	244	1.8	11	0.7	255	1.6	4	0.3	325	1.5	32	0.8	224	1.7	9	1.3
60–69	551	5.0	15	1.4	636	5.1	12	1.2	705	4.1	43	1.5	437	4.5	8	1.8
70–79	561	6.9	11	1.8	593	6.5	13	2.1	625	5.1	45	2.5	421	6.2	6	2.3
≥80	158	3.5	2	0.7	168	3.2	4	1.4	178	2.8	13	1.4	112	3.1	0	0.0
All ages†	1591	1.2	48	0.4	1743	1.1	35	0.3	1946	0.9	145	0.4	1260	1.0	26	0.4

* Deaths obtained from the multiple cause-of-death data for 1979–1998 are based on ICD-9 codes, and deaths beginning in 1999 are based on ICD-10 codes with available computerized literal death certificate data. Death information was also obtained from other surveillance mechanisms. Death rates expressed per 1,000,000. One death missing is both race and region; and one death is missing region.

† Death rates are age-adjusted.

### Variant Creutzfeldt-Jakob Disease

Since 1996, CDC received reports of three subsequently confirmed cases of vCJD in US residents [30]. The first of these vCJD case-patients was born in the United Kingdom in late 1979, moved to Florida in 1992, and experienced onset of vCJD symptoms in November 2001 at 22 years of age [13]. In January 2002, the patient returned to the United Kingdom for medical follow-up. The diagnosis of vCJD was initially made based on clinical symptoms, a characteristic “pulvinar sign” on magnetic resonance imaging (MRI) of the brain, and the presence of prion protein in tissue from a tonsil biopsy. The patient died in 2004 in the United States where neuropathological evaluation of brain tissue at autopsy provided confirmation of the vCJD diagnosis.

The second US resident with vCJD was also born in the United Kingdom and lived there throughout the defined period of increased risk for human exposures to the BSE agent, 1980–1996 [30]. The patient resided in the United States from 2001–2005 and experienced onset of symptoms in early 2005 at 30 years of age. The patient then returned to the United Kingdom where the diagnosis of vCJD was made based on symptoms and the presence of the “pulvinar sign” on brain MRI. The patient died in 2006 in the United Kingdom where vCJD was confirmed by neuropathological testing of brain tissue.

The third US resident with vCJD was born and raised in Saudi Arabia and beginning in 2001 he occasionally stayed in the United States for periods of up to 3 months duration [30], [31]. The patient relocated to the United States in 2005 where onset of vCJD symptoms was experienced in the spring of 2006. The diagnosis of vCJD was confirmed based on pathological study of adenoid and brain biopsy tissues in November 2006. The patient died later in 2006. The patient had no past history of neurosurgical procedures or visits to European countries. A previous case of vCJD attributed to consumption of BSE-contaminated cattle products had been reported in a Saudi Arabian resident [13].

## Discussion

The US incidence of CJD during 1979 through 2006 remained relatively stable at approximately 1 case per 1,000,000 persons; this incidence is similar to that reported in many other countries [23], [32]–[34]. In the present study, the large number of cases identified in ongoing US surveillance provides insights into the incidence of CJD by sex. These data demonstrate a majority (52.6%) of cases in females largely because of the higher number of women compared to men in the older age populations that experienced the highest CJD incidence rates. Incidence rates of CJD by sex were almost the same among persons <60 years of age and were higher among males relative to females as the age of the population groups increased. These findings are consistent with other studies that indicate a relative excess of cases among females [33], [35]–[38] but a higher incidence of CJD among the male population [35], [39]. The CJD incidence rates varied regionally, with the highest rate in the Northeast region and the lowest rates in the South and West regions. The low rate in the West is of particular interest due to the longstanding presence of chronic wasting disease (CWD) among cervids in parts of the region, particularly in Colorado and Wyoming.

Ongoing US surveillance data continue to demonstrate marked differences in CJD incidence by race and age. Similar to previous US reports [23], [35], [40], the age-adjusted incidence remains more than 2.5 times higher for whites than for blacks. Although the reasons for this disparity are unknown, it is possible that genetic differences and/or under-diagnosis among non-white patients could contribute. These continuing differences in incidence by race in the United States raise the possibility that the CJD incidence in countries where black populations predominate might be significantly lower than in countries where white populations predominate, an issue that deserves future study.

The incidence of CJD by age continues to show a pattern that is strikingly different from that reported for vCJD. Almost all vCJD cases, including all three US resident cases, died before 55 years of age. The US surveillance analysis illustrates that only about 11 percent of the CJD cases occurred in this younger US population. This relatively low proportion, and incidence rate, of CJD cases in this younger age group occurred despite an increased focus in US surveillance efforts on suspected cases in this age group since 1996 [12]. Cases of CJD <30 years of age in the United States remain extremely rare, with most of these cases being attributable to iatrogenic exposure or a genetic mutation.

The incidence of CJD dramatically increased with increasing age until it peaked in the 70–79 year age group. An explanation for the subsequent drop in incidence of CJD among those 80 years of age and older, a phenomenon consistently observed in earlier surveillance studies of CJD [40], remains uncertain. The rarity of CJD, however, in postmortem studies of possibly clinically unrecognized cases of CJD in the elderly has led to the suggestion that the decline in incidence among the elderly is real and unlikely an artifact of a varying sensitivity, by age group, of surveillance [41].

Since vCJD was initially recognized in the United Kingdom in 1996, CDC received reports of three subsequently confirmed cases of vCJD among US residents. The epidemiological data indicated that each of these cases was most likely infected in the United Kingdom (2 cases) and in Saudi Arabia (1 case) [13], [30].

Through mid-2009, twenty cases of BSE were identified among cattle in North America, including three that were identified in the United States [42]. In comparison, the United Kingdom reported more than 184,000 BSE cases as of September 30, 2008 and 168 cases of vCJD as of March 2, 2009 [16], [43], [44]. These BSE/vCJD data suggest that the greatest risk of vCJD in US residents will continue to be among persons who as a child or young adult consumed UK beef products during 1980–1996, the years when such products were most subject to BSE contamination [12], [30]. Such US residents would include those who were born and raised either in the United Kingdom or in another country where potentially BSE contaminated UK beef products were available for consumption. These persons would also include those who consumed such UK beef products as a child or young adult during visits abroad. The United States historically has imported few or no live cattle, beef products, or livestock nutritional supplements from the United Kingdom, and throughout the 1990s had banned the import of live ruminants and most ruminant products from known BSE countries [16]. Because indigenous BSE cases in North America were initially documented in 2003 and have continued to occur through 2008, albeit in relatively low numbers [42], the results of ongoing US vCJD surveillance receives considerable attention, particularly among those concerned about the emergence of indigenous vCJD cases in the United States.

Limitations of using the US national multiple cause-of-death data include possible coding and reporting discrepancies, and misdiagnosis of CJD as a cause of death. However, the use of national death certificate data has been found to be a reasonably sensitive (≥80%) method compared to more active methods of CJD case ascertainment [45], [46]. Furthermore, the US prion disease surveillance includes activities carried out by the NPDPSC. The NPDPSC, established by CDC in collaboration with the American Association of Neuropathologists, makes prion disease testing available free-of-charge to US physicians that can help to improve the accuracy of prion disease diagnoses [2], [47]. This center can confirm or refute the presence of vCJD, and also detect other unusual or new prion diseases [48]. The prion surveillance efforts further utilizes the laboratory test results in the investigation and identification of CJD among persons <55 years of age. Finally, the use of the SuperMICAR procedure beginning in 1999 (fully in 2003) enables detection of deaths with prion disease even if cause of death on the death certificate is miscoded (e.g., an inappropriate coding rule, a misinterpretation or a misreading of the certificate) [22].

The occurrence of CJD and vCJD continues to be an international and national concern. Ongoing CJD and vCJD surveillance in many countries of the world, including the United States, remains critical for determining the extent to which the agents of classic and possibly atypical BSE may cause disease in humans [49]. Physicians and health care workers in the United States are encouraged to indicate CJD, as appropriate, on death certificates for all their patients who die with CJD or vCJD. In addition, health care workers who provide care to patients with suspected or clinically diagnosed CJD or vCJD should discuss possible options for autopsy with their local and state health department and the NPDPSC. Brain tissue specimens obtained by autopsy from these patients may be submitted to the NPDPSC for further analysis and confirmation of the CJD diagnosis. The ongoing prion disease surveillance and diagnostic testing is important for monitoring the stability of the CJD incidence rates, and detecting possible occurrences of vCJD and other new prion diseases in the United States.
